# Three doses of Sars-CoV-2 mRNA vaccine in older adults result in similar antibody responses but reduced cellular cytokine responses relative to younger adults

**DOI:** 10.1016/j.jvacx.2024.100564

**Published:** 2024-09-25

**Authors:** Geir Bredholt, Marianne Sævik, Hanne Søyland, Thor Ueland, Fan Zhou, Rishi Pathirana, Anders Madsen, Juha Vahokoski, Sarah Lartey, Bente E. Halvorsen, Tuva B. Dahl, Mai-Chi Trieu, Kristin G.-I. Mohn, Karl Albert Brokstad, Pål Aukrust, Camilla Tøndel, Nina Langeland, Bjørn Blomberg, Rebecca Jane Cox, Bergen COVID-19 Research Group

**Affiliations:** a Department of Clinical Science, University of Bergen, Bergen, Norway; b Influenza Centre, Department of Clinical Science, University of Bergen, Bergen, Norway; c Department of Medicine, Haukeland University Hospital, Bergen, Norway; d Faculty of Medicine, Institute of Clinical Medicine, University of Oslo, Oslo, Norway; e Research Institute of Internal Medicine, Oslo University Hospital Rikshospitalet, Oslo, Norway; f Department of Microbiology, Haukeland University Hospital, Bergen, Norway; g Department of Safety, Chemistry and Biomedical Laboratory Sciences, Western Norway University of Applied Sciences, Bergen, Norway; h Section of Clinical Immunology and Infectious Diseases, Oslo University Hospital, Oslo, Norway; i Department of Pediatrics, Haukeland University Hospital, Bergen, Norway; j National Centre for Tropical Infectious Diseases, Department of Medicine, Haukeland University Hospital, Bergen, Norway

**Keywords:** SARS-CoV-2, mRNA vaccines, Aging, Neutralizing antibodies, Memory B cells, T-lymphocytes, Interferon-gamma

## Abstract

**Objectives:**

Booster COVID-19 vaccinations are used to protect the elderly, a group vulnerable to severe disease. We compared humoral and cellular immunity in older versus younger adults up to eight months after administering a BNT16b2 booster vaccine dose. Next, we analyzed the plasma levels of soluble T cell activation/exhaustion markers.

**Methods:**

Home-dwelling older adults (n = 68, median age 86) and younger healthcare workers (n = 35, median age 39), previously vaccinated with two doses of BNT162b2, were given a booster dose at ten months after the initial dose. Our analysis consisted of spike-specific IgG, neutralizing antibodies, memory B cells, IFN-γ and IL-2 secreting T cells and soluble T cell exhaustion/activation markers.

**Results:**

Following the initial two doses, the elderly cohort exhibited lower humoral and IFN-γ responses compared to younger adults. The booster dose increased the humoral responses in both older and younger adults. At two months after the booster dose, older and younger vaccinees had comparable levels of antibodies and the responses were maintained up to 18 months. The younger cohort elicited an increase in the cellular response, while no increase was detected in the elderly. The elderly had higher plasma levels of soluble forms of the T cell activation/exhaustion markers CD25 and TIM-3, which inversely correlated with age and T-cell cytokine responses. This suggests that these markers may be related to the observed dysfunctional cellular cytokine response in older adults. However, both elderly and younger adults who experienced breakthrough infections after booster vaccination, elicited more robust humoral and IFN-γ responses.

**Conclusions:**

The booster dose elicited neutralizing and spike-specific antibody responses in the elderly that were comparable with that of the younger cohort. However, the lack of a strong cellular cytokine response to the third dose in the elderly may explain their vulnerability to severe infection and may be a consequence of exhausted or senescent T cell responses. (https://clinicaltrials.gov/study/NCT04706390).

## Highlights

• No increase in cytokine producing T cells was observed after the booster vaccination in the elderly.
• The elderly had higher plasma levels of soluble T-cell activation/exhaustion markers that correlated inversely with age and T-cell responses.
• Booster vaccination was needed in older adults to increase memory B cells to the SARS-CoV-2 receptor binding domain.
• SARS-CoV-2 mRNA vaccine booster increases antibody response to comparable levels in older and younger adults.
• Antibody responses were maintained for eight months after boosting in both groups.

## Introduction

The SARS-CoV-2 BNT162b2 mRNA vaccine is one of the most extensively used COVID-19 vaccines worldwide. The primary vaccination schedule consists of two doses administered 21 days apart [1], but subsequent studies have indicated that extending the interval between doses can enhance the immunogenicity of this vaccine [2], [3]. Moreover, the efficacy of the BNT162b2 vaccine against severe disease, hospitalization and death have shown a notable decrease approximately six months after primary vaccination, likely attributed to waning of vaccine-induced immunity [4], [5]. Booster vaccination regimens have been employed to enhance the immune responses and restore vaccine effectiveness to levels comparable to or higher than that observed after primary vaccination [6], [7]. However**,** questions persist regarding discrepancies in vaccine-induced immune responses, particularly in some age groups. Especially, the kinetics, magnitude and duration of the adaptive immune response in older individuals, who typically have poorer vaccine immunogenicity and more co-morbidities, are still not fully understood. A substantial body of evidence indicates that COVID-19 vaccines are significantly less immunogenic in the older population compared to younger adults following primary vaccination [8], [9], [10], [11]. These differences have been attributed to various factors, including immunosenescence and underlying health conditions [10], [12]. Some studies indicate that a third dose can help bridge the immunogenicity gap between the older and younger populations [13], [14], while other studies suggest even more doses to bridge this gap [15].

In a previous study we reported the kinetics and durability of the humoral and cellular immune responses in older adults (median age 86) and younger individuals (median age 38) following two doses of the BNT162b2 vaccine for up to nine months [16]. In comparison to the younger cohort, the older adults had lower anti spike-IgG, neutralizing antibody, spike-specific memory B cells (MBC) and spike-specific breadth and depth of the CD4+ and CD8+ T cell receptor repertoires. The current study extends this investigation to assess the immune responses in both cohorts after a third dose (booster dose) and up to 18 months post-vaccination. Additionally, we examined the immune responses in some individuals who experienced breakthrough COVID-19 infection. Our data present a mixed perspective regarding the immunogenicity of the booster vaccine dose in older and younger cohorts. While the spike binding IgG and neutralizing antibody levels were comparable between the cohorts, the third dose elicited a T cell response primarily in the younger cohort. The failure to induce long-term, functional T cellular responses suggests that the elderly populations may remain at a higher risk of severe COVID-19 despite receiving repeated vaccinations, emphasizing the need for tailored vaccines in this age group.

## Materials and methods

### Study population

We have previously conducted a prospective comparative cohort study of younger adults and home dwelling older adults up to nine months after receiving two doses of the COVID-19 mRNA vaccine (BNT162b2 Pfizer-BioNTech) [16]. The participants for the current, extended time study included data for two and eight months after booster vaccination (12 and 18 months after first dose) and included SARS-CoV-2 breakthrough infection history.

The older adults were recruited through a general practice, and the younger adults were health care workers recruited at Haukeland University Hospital, both located in Bergen, Norway. The study was conducted according to the guidelines of the Declaration of Helsinki and was approved by the Regional Committee for Medical Research Ethics, Northern Norway (REK Nord number 218629) and is registered in the National Institute for Health database Clinical trials.gov (NCT04706390). All participants signed written, informed consent before inclusion in the study. The inclusion criteria were eligibility for COVID-19 vaccination and willingness to attend scheduled blood sampling visits while the exclusion criteria were history of hypersensitivity or anaphylaxis after vaccination.

Participants were followed clinically and immunologically along with blood sampling at baseline (D0), at 3 and 6 weeks, and at 5, 9, 12 and 18 months after the first vaccination.

### Vaccines

Both BNT162b2 (COMIRNATY, Pfizer-BioNTech) and mRNA-1273 (Spikevax, Moderna) consist of single-stranded, 5′-capped, nucleoside-modified mRNA encoding a stabilized prefusion form of the spike glycoprotein of the SARS-CoV-2 Wuhan-Hu-1 strain (Genbank MN908947) [17], [18]. Both vaccines are lipid nanoparticle (LNP) formulated but with differences in lipid and mRNA composition, and mRNA dose [19].

### Study design

Demographic and clinical data were registered in electronic case report forms (eCRF), using the Research Electronic Data Capture tools (REDCap; Vanderbilt University, Nashville, TN, USA). Data included information on gender, age, medications including immunosuppressants, comorbidities, SARS-CoV-2 PCR test results, COVID-19 symptoms, and COVID-19 vaccination data.

All participants (n = 111) were vaccinated with two doses of 0.3 ml BNT162b2 (COMIRNATY, Pfizer/BioNTech, 30 µg mRNA) mRNA vaccine at 3-week intervals into the deltoid muscle during January-February 2021. Ten months after the first dose (November-December 2021) the remaining participants (n = 104) received an intramuscular booster dose; either as 0.3 ml BNT162b2 mRNA vaccine in most (n = 98) participants, or as 0.25 ml (half dose containing 50 µg mRNA) of the mRNA-1273 (Spikevax, Moderna) vaccine in a minority (n = 6) of the participants (median 294 vs 320 days since D0 for older vs younger adults).

Serum and plasma were collected at baseline (D0), at 3 and 6 weeks, and at 5, 9, 12 and 18 months after the first vaccination using clot activator tubes (CAT, BD, UK) and EDTA tubes (BD, UK), respectively. Samples were aliquoted and stored at −80 °C. Peripheral blood mononuclear cells (PBMCs) were isolated from a subgroup of the vaccination cohorts using Cell Preparation Tubes (CPT, BD, UK) according to the manufacturer's instructions. PBMCs were diluted in cell culture medium (RPMI-1640 with L-glutamine (Lonza), 10 % heat-inactivated fetal bovine serum (FBS, Hyclone), 100 U/ml penicillin and 0.1 mg/ml streptomycin (Sigma-Aldrich)) and used directly in cellular assays.

### Antigens and peptides

Purified SARS-CoV-2 (Wuhan-Hu-1 isolate) receptor binding domain (RBD) and spike proteins were produced in-house from constructs provided by Professor Florian Krammer and expressed and purified as previously described [20]. Libraries of synthetic peptides (17-mers, with 10 amino acid overlaps, > 80 % pure) of the full length of SARS-CoV-2 USA-WA1/2020 spike protein (S) and nucleocapsid protein (N) were obtained from BEI Resources (VA, USA). The peptides were solubilized in DMSO (≥ 99.9 %), pooled and diluted in cell culture medium to a final DMSO concentration of < 0.5 % [21]. To prevent toxic DMSO concentrations (> 0.5 % DMSO) the peptides for the S protein were combined in two distinct pools, S1 (a.a.1–689) covering the S1 domain and S2 (a.a.680–1273) covering the S2 domain. The N protein peptides were combined in one pool.

### Measurements of anti-spike IgG

The anti-spike IgG ELISA was performed as previously described [16], [22], except that all baseline sera were analyzed for SARS-CoV-2 spike IgG. Sera were serially diluted in a 5-fold manner from 1:100 in duplicate and detected using horseradish peroxidase (HRP)-labelled anti-human IgG (SouthernBiotech, Birmingham, AL, USA) followed with the chromogenic substrate 3,3́,5,5́-tetramethylbenzidine (TMB; BD Biosciences, San Jose, CA, USA). Optical density (OD) was measured at 450/620 nm using the Synergy H1 Hybrid Multi-Mode Reader with the Gen5 2.00 (version 2.00.18) software (BioTek Instruments Inc., Winooski, VT, USA) and anti-spike IgG endpoint titers were determined. Positive controls were serum from a hospitalized COVID-19 patient and the well characterized spike-specific monoclonal antibody CR3022, whereas pooled pre-pandemic sera (n = 128) were used as a negative control [22]. Samples with no detectable antibodies were assigned a titer of 50 for calculation purposes. Results were reported as geometric mean titers (GMT) or as individual titers.

### Microneutralization

Antibody neutralization titers were determined using a locally isolated SARS-CoV-2 D614G strain from March 2020 (hCoV-19/Norway/Bergen-01/2020, GenBank accession ID OM616023.1, GISAID accession ID EPI_ISL_541970) in a certified biosafety level 3 laboratory, as previously described [16]. Neutralization titers were determined as the reciprocal of the serum dilution giving 50 % inhibition of virus. Negative titers were assigned a value of 10, half of the starting dilution of 1/20, for calculation purposes. Results were reported as geometric mean titers (GMT) or as individual titers.

### Memory B cell ELISpot

Memory B cell ELISPOT analysis was performed as described previously using fresh PBMCs [16]. Memory B cells were polyclonally activated and expanded in 24 well plates (Thermo Fisher Scientific, Catalog # 142475) by incubating one million PBMCs from each blood sample in 1 ml cell culture medium containing 1 mg/ml R848 and 1 µg/ml recombinant human interleukin 2 (rhIL-2) (Mabtech AB, Sweden) for 6 days at 37 °C, 5 % CO_2_. One million PBMCs were incubated in medium alone as negative controls. ELISpot plates (MultiscreenHTS MSHAS4510, Millipore) were coated with 15 µg/ml anti-human IgG (MT91/145, Mabtech) in PBS, 10 µg/ml SARS-CoV-2 Spike protein in PBS, 10 µg/ml SARS-CoV-2 RBD in PBS and PBS only (uncoated negative control) at 4 °C overnight, washed and blocked with cell culture medium prior to use. Non-stimulated and stimulated lymphocytes were washed twice with medium and transferred in duplicate to the coated ELISpot plates and incubated undisturbed for 16 h (37 °C, 5 % CO_2_). Plates were washed and anti-Spike, anti-RBD and total IgG + memory B cells were detected with 1 µg/ml biotinylated anti-IgG mAbs (MT78/145, Mabtech) for 2 h at room temperature followed by Streptavidin-HRP (1:1000, Mabtech) for one hour. Spots were developed with 3,3′,5,5′ Tetramethylbenzidine (TMB) ELISpot substrate (Mabtech) according to manufacturer’s instructions. Plates were dried and counted using an ELISpot reader (Advanced Imaging Devices, Germany). Spike- and RBD specific spots were calculated as the mean of the duplicate wells after subtraction of negative control wells. Results are presented as spot forming units (SFU) per 1 × 10^6^ PBMCs.

### Interferon-γ and interleukin-2 dual FluoroSpot assay

Antigen-specific interferon-γ (IFN-γ) and IL-2 secreting T cells were quantified using FluoroSpot assay (Mabtech AB, Sweden) as described previously [21].

Briefly, 200,000 PBMCs/well were stimulated in duplicate with SARS-CoV-2 peptides (1 µg/mL), negative controls (DMSO, medium alone) and 50.000 PBMCs/well were stimulated in duplicate with anti-CD3 antibody (positive control). Plates were incubated for 16 h (37 °C, 5 % CO_2_) and developed according to the manufacturer’s instructions. The average spot forming units (SFU) of duplicates were counted using a fluorescence reader fitted with color filters for FITC and Cy3 (Advanced Imaging Devices, Germany) and background from negative controls were subtracted. Results are presented as SFU per 1 × 10^6^ PBMCs.

### Measurements of soluble markers of T cell activation/exhaustion

Plasma levels of soluble (s)CD25, soluble carcinoembryonic antigen-related cell adhesion molecule 1 (sCEACAM1), soluble T cell immunoglobulin and mucin-domain containing-3 (sTIM-3) and soluble lymphocyte antigen (sLAG)-3 were measured in duplicate by enzyme-linked immunosorbent assay (ELISA) using commercially available antibodies (R&D Systems, Minneapolis, MN) in a 384-format using a combination of a SELMA pipetting robot (Analytik Jena AG, Jena, Germany) and a BioTek dispenser/washer (BioTek Instruments, Winooski, VT). Samples were thawed only once. Absorption was read at 450 nm by using an EIA plate reader (BioTek Instruments) with wavelength correction set to 540 nm. Samples and controls were run on the same 384-well plate.

### Statistics

GraphPad Prism (Version 10.0.3; La Jolla, CA, USA) was used to analyze data and generate figures. Statistical comparisons were evaluated using unpaired, nonparametric Kruskal-Wallis test with Dunn's multiple comparisons test. Spearman correlation was used to assess correlations.

## Results

### Study groups and demographics

**Fig. 1:**
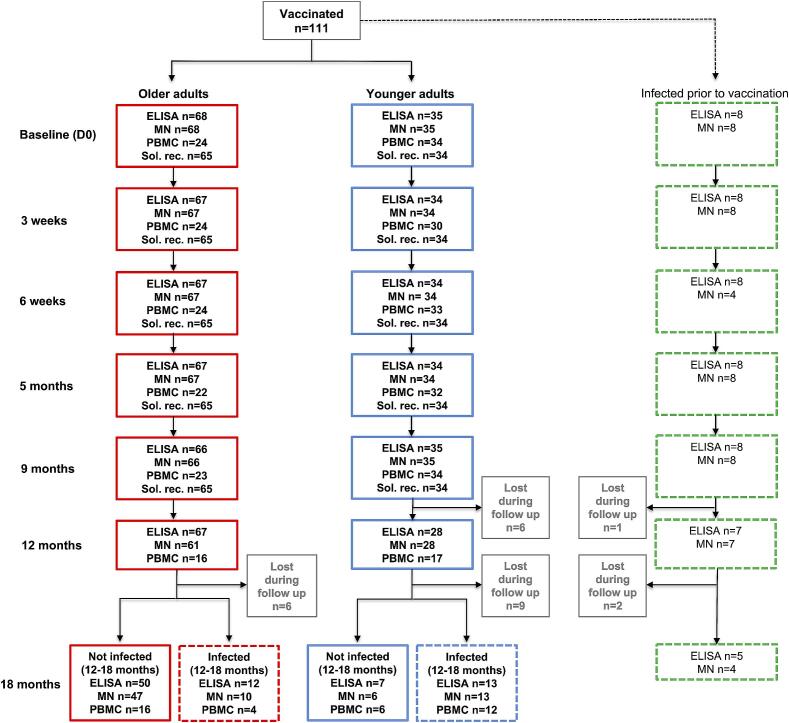
**Study population flowchart.** Number of vaccinees included at baseline and number of vaccinees analyzed with serological assays (ELISA, microneutralization (MN), cellular assays (peripheral blood mononuclear cells (PBMC)) and assay for soluble T cell activation/exhaustion receptors (Sol. rec.) in plasma during the study.

The older vaccinees consisted of 68 home-dwelling elderly (73–98 years old (yo), median 86 yo, 57 % females), all of whom had no reported SARS-CoV-2 positive PCR test and were seronegative prior to vaccination (all MN titers 10) (Fig. 1, Table 1). The younger vaccinees consisted of 35 health care workers (27–63 yo, median 39 yo, 66 % females) with no reported SARS-CoV-2 positive PCR test and were seronegative prior to vaccination (MN titers 10–24, GMT 10). The elderly had more comorbidities (87 %) compared to younger adults (6 %) (Table 1). Eighteen percent of the elderly were taking immunosuppressive medication, compared to 3 % of the younger adults.

**Table 1:** Demographics of the study population.

	Older adults	Younger adults	Infected prior to vaccination
n	68	35	8
Median Age (range)	86 (73–98)	39 (27–63)	46 (27–87)
Females	39 (57 %)	23 (66 %)	2 (25 %)
Comorbidities*	59 (87 %)	2 (6 %)	2 (25 %)
Chronic heart disease	49 (72.1 %)	0	2 (25 %)
Chronic lung disease	10 (14.7 %)	1 (2.9 %)	0
Rheumatic disease	9 (13.2 %)	0	0
Diabetes	6 (8.8 %)	0	0
Cancer	6 (8.8 %)	0	0
Chronic liver disease	5 (7.4 %)	0	0
Chronic kidney disease	3 (4.4 %)	0	0
Neurological disease	3 (4.4 %)	0	0
Autoimmune disease	1 (1.5)	1 (2.9 %)	0
Immunosuppression^1^	12 (18 %)	1 (2.9 %)	2 (25 %)

1 Prednisolone or other immunosuppressive medication (fluorouracil, potassium folinate, irinotecan, rituximab or hydroxyurea).

Older (n = 12) and younger (n = 13) participants that were infected with SARS-CoV-2 between 12 and 18 months during the first omicron pandemic wave were separated into individual groups at 18 months. Infections were confirmed by SARS-CoV-2 positive PCR (n = 18), and/or a positive IFN-γ T cell FluoroSpot to SARS-CoV-2 nucleocapsid protein (n = 12, range 40–265, median 60 SFU/million PBMC)), and/or a ≥ 2-fold increase in MN titers between 12 and 18 months (n = 17, range 2–123 fold increase, median 3-fold increase). None of the individuals with breakthrough infections had severe disease requiring hospitalization. The median time from positive PCR test to 18 months sampling was 122.0 days in the six older adults (range 93–168 days) and 101.5 days in the 13 younger adults (range 76–139 days).

In addition, we collected sera from a small cohort of eight vaccinated COVID-19 convalescents irrespective of age (27–87 yo, median 46 yo, 25 % females) consisting of two older adults both 87 years old and six health care workers 27–61 yo (median 37 yo) as determined by previous SARS-CoV-2 positive PCR test (n = 6) and/or a pre-vaccination MN titer > 30 (n = 7, 10–1265, GMT 110). The median time from positive PCR test to first vaccine dose for the six individuals was 210 days (range 53–317 days).

### Serological responses

#### Spike specific IgG

**Fig. 2:**
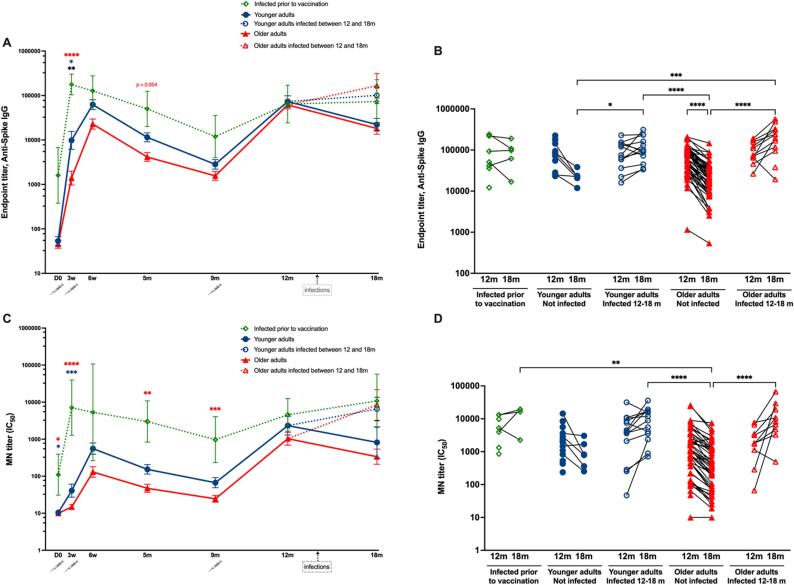
**Kinetics of SARS CoV-2 specific serum antibody responses after vaccination and boosting.** (A) Anti-spike IgG endpoint titers measured by ELISA in younger adults, older adults and individuals recovered from SARS-CoV-2 infection before vaccination. (B) Individual anti-spike IgG ELISA endpoint titers at 12 and 18 months in younger and older adults. (C) Neutralizing antibody titers measured by microneutralization assay (MN) against ancestral D614G strain in younger adults, older adults, and pre-vaccination convalescents. (D) Individual MN titers at 12 and 18 months in younger adults, older adults, pre-vaccination convalescents and younger and older adults infected with SARS-CoV-2 between 12 and 18 months. Data are presented as geometric mean titers (GMT) with 95 % confidence intervals (A, C) or as individual titers (B, D). Blue and red dotted lines with open symbols represent younger and older adults with breakthrough infections between 12 and 18 months, respectively. Significant differences were assessed by Kruskal-Wallis and Dunn’s multiple comparisons test. ****P < 0.0001, ***P < 0.001, **P < 0.01, *P < 0.05. Black, blue and red asterisks in figure A and C denote significant differences between younger versus older adults, pre-vaccination convalescents versus younger adults, and pre-vaccination convalescents versus older adults, respectively.

**Supplementary Fig. 1:**
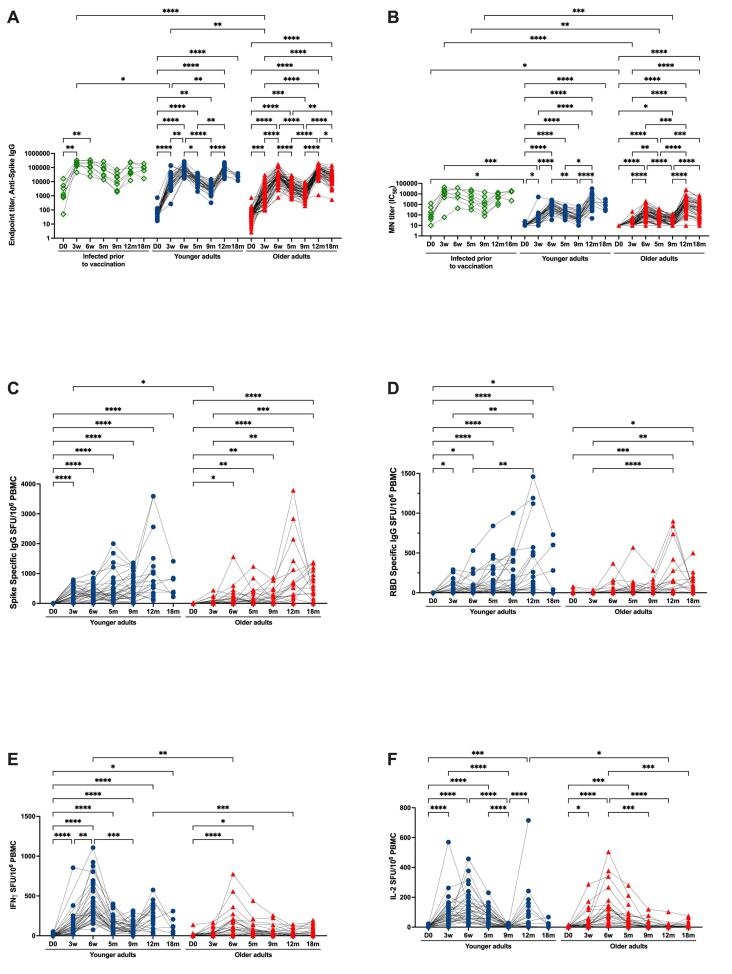
**Individual humoral and cellular responses in younger and older adults at 0–18 months**. (A) Individual spike specific IgG endpoint titers measured by ELISA in younger adults, older adults and individuals recovered from SARS-CoV-2 infection before vaccination. (B) Individual microneutralization antibody titers against ancestral D614G strain in younger adults, older adults, and pre-vaccination convalescents. (C) Individual spike-specific IgG memory B cells (MBC) measured by ELISpot in younger and older adults. Data are presented as spot forming cells/units (SFU) per million PBMC. (D) Individual RBD-specific IgG memory B cells (MBC) measured by ELISpot in younger and older adults. Data are presented as SFU per million PBMC (peripheral blood mononuclear cells). (E) Individual IFN-γ secreting T-cells in younger and older adults. Data are presented as SFU per million PBMC. (F) Individual IL-2 secreting T-cells in younger and older adults. Data are presented as SFU per million PBMC. Significant differences were assessed by Kruskal-Wallis and Dunn’s multiple comparisons test. ^****^P < 0.0001, ^***^P < 0.001, ^**^P < 0.01, *P < 0.05. Individuals with breakthrough infections between 12 and 18 months are not included at 18 months.

Briefly, the first vaccination induced a significant increase in IgG to the spike protein in all three groups at three weeks, significantly higher in previously infected individuals and significantly lower in the older adults (Fig. 2 and supplementary Fig. 1A). The second vaccine dose boosted the spike-specific IgG titers especially in older adults at six weeks, but not in previously infected individuals. After five months, the antibody titers had decreased in older and younger adults compared to six weeks and declining further at nine months. Antibody titers also decreased successively in the previously infected individuals at five and nine months.

The booster vaccination (approximately 10 months post primary vaccination) increased antibody responses in older and younger adults and previously infected individuals (GMT 61578, 73461 and 64248, respectively). Between 12 and 18 months there was a significant decrease in the antibody titers in non-infected older adults although remaining significantly higher compared to 5 and 9 months, thus suggesting the booster dose improved the antibody titers for a prolonged period. There was no significant difference in antibody titers between non-infected younger and older adults after boosting at 12 and 18 months. The titers at 18 months for the older and younger adults infected between 12 and 18 months were significantly higher compared to their non-infected counterparts (Fig. 2B).

### Microneutralization

Prior to vaccination the MN titers were significantly higher in the previously infected group compared to older and younger adults (Fig. 2C and supplementary Fig. 1B). Primary vaccination significantly increased MN titers in younger adults and older adults. At five months, MN titers decreased in the older and younger adults and previously infected individuals, declining further at nine months to significantly lower titers compared to six weeks in both older and younger adults. No significant differences in MN titers between the older adults and younger adults were observed at any time point up to and including nine months.

Two months after the 10-month booster vaccination, the MN titers increased in both the older and younger adults (GMT 1031 and 2320) to the highest titers with a 41- and 35-fold increase in GMT above pre-booster levels, respectively. Between 12 and 18 months, the MN titers decreased in the non-infected older and younger adults although they were maintained between 12 and 18 months in previously infected individuals. In the older and younger adults with breakthrough infections between 12 and 18 months, there was a non-significant trend towards an increase in MN titers (Fig. 2D). The older adults with breakthrough infections had significantly higher MN titers at 18 months compared to their non-infected counterparts (Fig. 2D).

### Cellular immune responses

We further investigated the cellular responses in a naïve subgroup of 24 older adults (73–93 years old (yo), median 86 yo, 63 % females) and 34 younger adults (27–63 yo, median 39 yo, 65 % females).

### Spike- and RBD-specific IgG Memory B cells

**Fig. 3:**
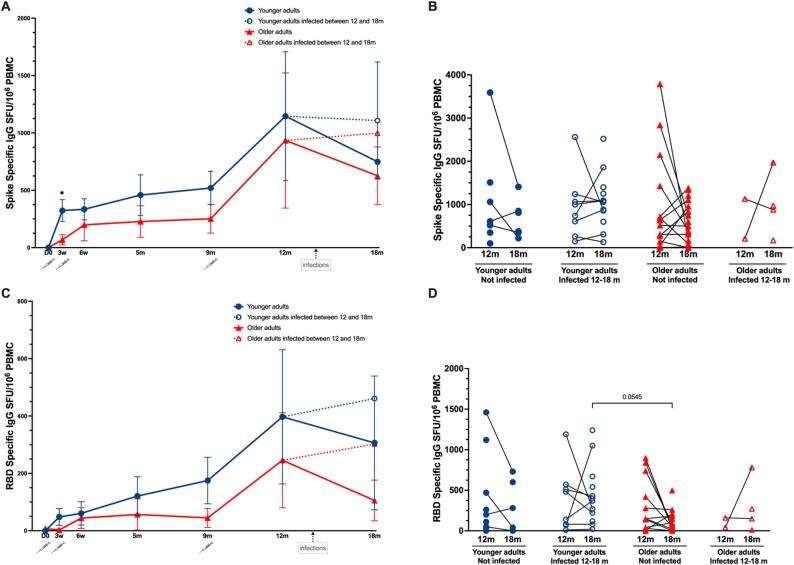
**SARS CoV-2 specific IgG memory B cell responses after vaccination and boosting.** (A) Spike-specific IgG memory B cells (MBC) measured by ELISpot in younger and older adults. (B) Individual anti-spike IgG MBC at 12 and 18 months in younger and older adults, and younger and older adults infected with SARS-CoV-2 between 12 and 18 months. (C) SARS-CoV-2 RBD-specific IgG MBC measured by ELISpot in younger and older adults. (D) Individual RBD-specific IgG MBC at 12 and 18 months in younger adults, older adults, and younger and older adults infected with SARS-CoV-2 between 12 and 18 months. Data are presented as mean spot forming cells/units (SFU) per million PBMC with 95 % confidence intervals (A, C) or as individual SFU per million PBMC (B, D). Blue and red dotted lines with open symbols represent younger and older adults with breakthrough infections between 12 and 18 months, respectively. Significant differences were assessed by Kruskal-Wallis and Dunn’s multiple comparisons test. *P < 0.05.

Prior to vaccination no spike- or RBD-specific MBC were detected, indicating that the participants were primarily immunologically naïve to SARS-CoV-2 (Fig. 3 A, C and supplementary Fig. 1 C, D). At three weeks, there was a significant increase in spike and RBD specific MBC frequencies in the younger adults, but not in the older adults. Between three and six weeks the MBC frequencies to spike increased significantly in both groups compared to pre-vaccination MBC levels. In the older adults the RBD-specific MBC did not increase significantly after two vaccine doses. Between six weeks and nine months the frequencies of spike protein and RBD-specific MBC were maintained in both subgroups.

The booster vaccination between nine and 12 months increased the frequencies of spike and RBD-specific MBC at 12 months in the younger and older adults. The increase of RBD-specific MBC was most prominent among the younger adults where the levels at 12 months were significantly higher compared to six weeks. Notably, the older adults needed booster vaccination to significantly increase frequencies of RBD specific MBC compared to prevaccination frequencies (supplementary Fig. 1 D).

In the older and younger adults with breakthrough infections between 12 and 18 months, the frequencies of Spike and RBD specific MBC were maintained at 18 months whereas MBC frequencies decreased in the non-infected older and younger adults, although not significantly. There was no significant difference in spike or RBD specific MBC between non-infected younger and older adults after boosting at 12 and 18 months. The MBC levels at 18 months for the older and younger adults infected between 12 and 18 months were significantly higher compared to their non-infected counterparts (Fig. 3B and 3D).

### Spike specific IFN-γ and IL-2 T cell responses

**Fig. 4:**
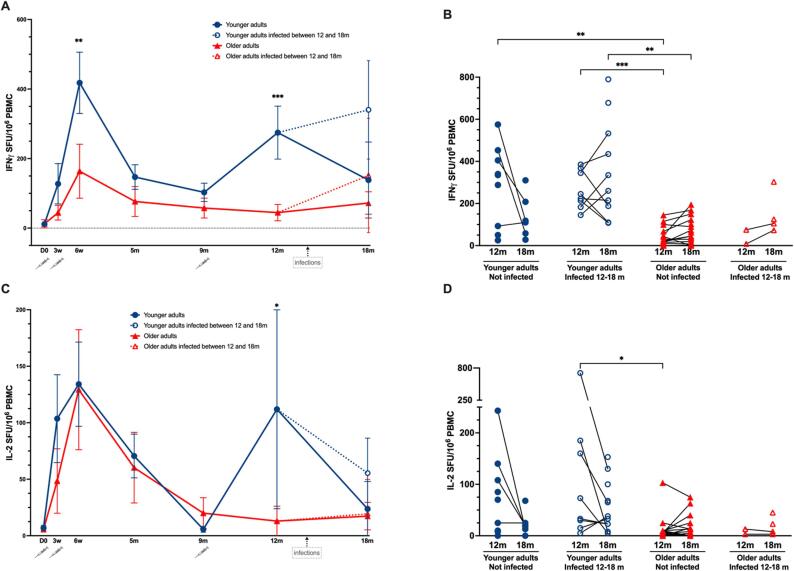
**SARS CoV-2 spike specific T-cell responses after vaccination and boosting.** (A) IFN-γ secreting T cells measured by FluoroSpot in younger and older adults. (B) Individual IFN-γ secreting T-cells at 12 and 18 months in younger and older adults, and younger and older adults infected with SARS-CoV-2 between 12 and 18 months. (C) IL-2 secreting T-cells measured by FluoroSpot in younger and older adults. (D) Individual IL-2 secreting T-cells at 12 and 18 months in younger and older adults, and younger and older adults infected with SARS-CoV-2 between 12 and 18 months. Data are presented as mean spot forming cells/units (SFU) per million PBMC with 95 % confidence intervals (A, C) or as individual SFU per million PBMC (B, D). Blue and red dotted lines with open symbols represent younger and older adults with breakthrough infections between 12 and 18 months. Significant differences were assessed by Kruskal-Wallis and Dunn’s multiple comparisons test. ***P < 0.001, **P < 0.01, *P < 0.05.

**Supplementary Fig. 2:**
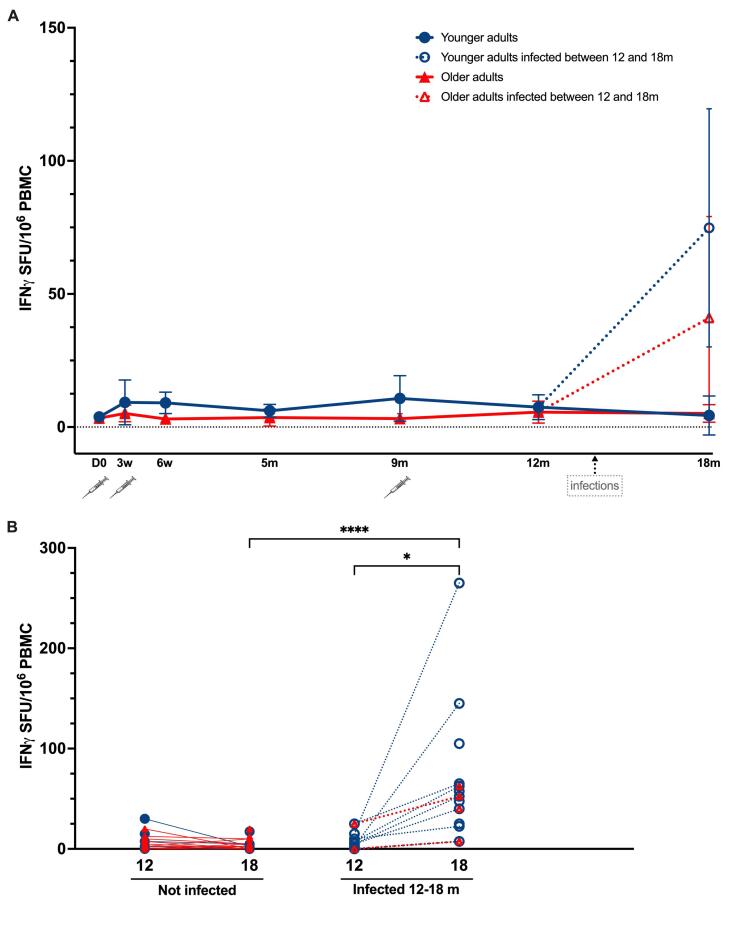
**SARS CoV-2 nucleocapsid specific T-cell responses to SARS-CoV-2 spike peptide pool after vaccination and boosting**. Number of IFN-γ producing cells in response to SARS-CoV-2 nucleocapsid peptide pool day 0 to 18 months in younger adults, older adults, and younger and older adults infected with SARS-CoV-2 between 12 and 18 months. Data are presented as mean spot forming cells/units (SFU) per million PBMC with 95% CIs (A) or as individual responses at 12 and 18 months of vaccinees not infected between 12 and 18 months versus vaccinees with breakthrough infections between 12 and 18 months (B). Blue and red dotted lines with open symbols represent younger and older adults with breakthrough infections between 12 and 18 months. Significant differences were assessed by Kruskal-Wallis and Dunn’s multiple comparisons test. ^****^P < 0.0001, *P < 0.05.

Pre vaccination, no IFN-γ and IL-2 producing cells were detected, except in an older and a younger adult (Fig. 4A and supplementary Fig. 1E) probably due to cross-reactive spike T cells. Neither of these individuals had pre-vaccination positive PCR, spike-specific antibody titers or MBC, or nucleocapsid specific T cells (supplementary Fig. 2) At three weeks post-vaccination, there was a significant increase in IFN-γ producing cells in the younger adults but not the older adults (Fig. 4A and supplementary Fig. 1E). In contrast, IL-2 producing cells significantly increased by three weeks in both older and younger participants (Fig. 4C and supplementary Fig. 1F). At six weeks, the frequencies of IFN-γ producing cells significantly increased in the younger adults compared to three weeks.

At nine months, the frequencies of IFN-γ producing cells decreased significantly (compared with 6 weeks) only in the younger adults. During the same interval, IL-2 levels also decreased significantly to low levels in both groups.

Interestingly, at 12 months (two months after the booster dose) frequencies of IFN-γ and IL-2 producing cells increased in most of the younger adults, while most of the older adults failed to mount a response. At 12 months the younger adults had significantly higher numbers of IFN-γ and IL-2 producing cells compared to the older adults. Between 12 and 18 months there was a trend of decreasing IFN-γ producing T cells in the non-infected younger adults, while in the noninfected IFN-γ producing T cell responses in older adults were maintained. Vaccinees who experienced breakthrough infection had in general an increase in number of nucleocapsid specific IFN-γ secreting T cells at 18 months (supplementary Fig. 2).

When looking at the individual responses, we found that four (17 %) of the older adults failed to produce IFN-γ producing cells above detectable levels from six weeks and throughout the study period. Fifteen (87 %) of the older adults and nine (26 %) of the younger adults did not maintain IFN-γ producing cells above detectable levels from six weeks and throughout the study period.

### Soluble markers of T cell activation/exhaustion

**Fig. 5:**
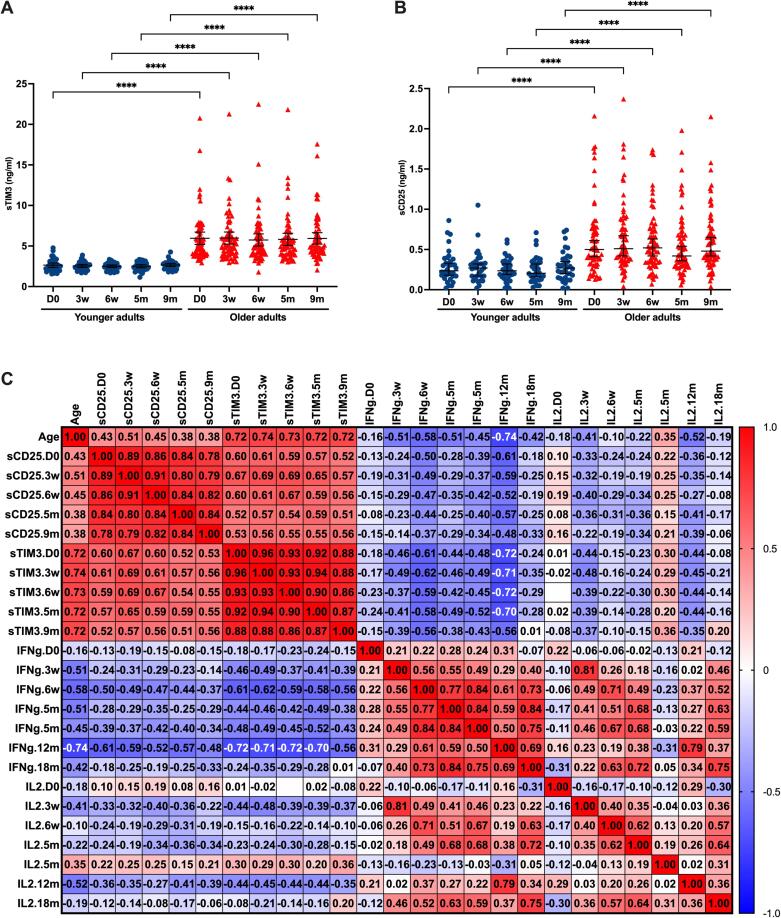
**Associations between soluble T cell activation/exhaustion markers (sCD25, sTIM-3), age, and IFN-γ/IL-2 responses to spike peptides.** Plasma levels of sTIM-3 (A) and sCD25 (B) from baseline to 9 months post vaccination in younger adults and older adults. Significant differences were assessed by Kruskal-Wallis and Dunn’s multiple comparisons test. ****P < 0.0001. (C) Spearman correlation matrix between age, sCD25, sTIM-3, and IFN-γ (IFNg) and IL-2 (IL2) secreting cells to the spike protein peptide pool at different sampling time points. For the 18 months data, individuals infected with SARS-CoV-2 between 12 and 18 months are not included. Spearman correlation coefficients (r) are shown.

**Supplementary Fig. 3:**
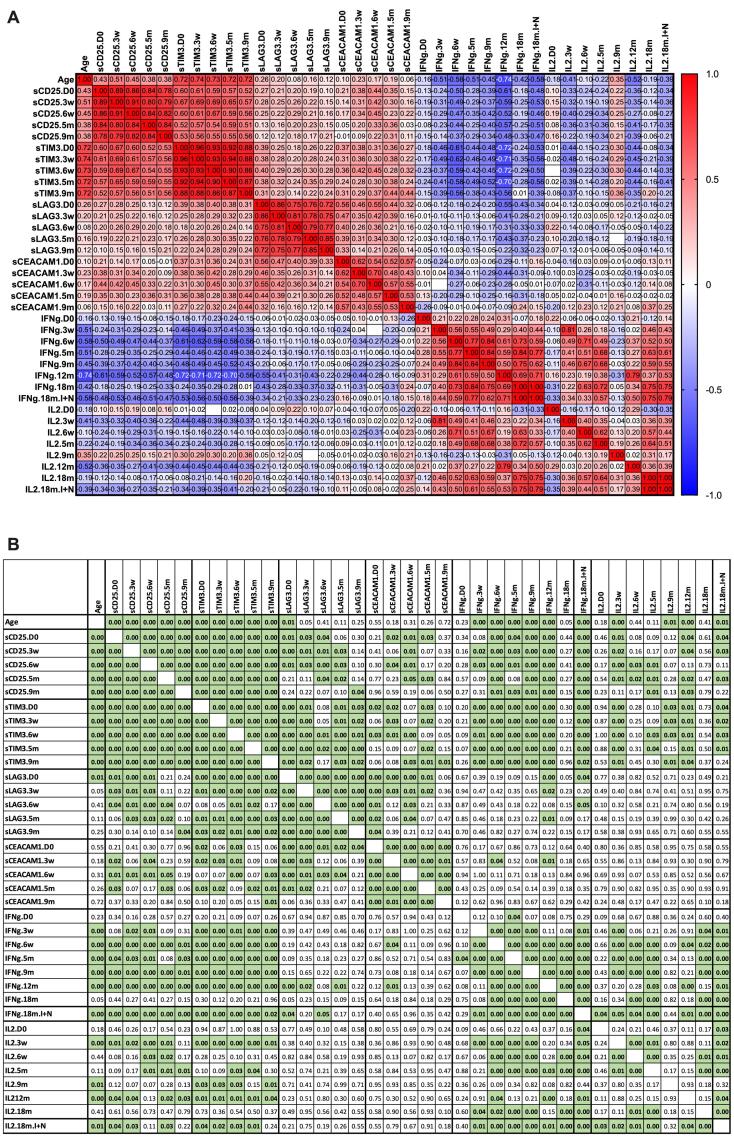
**Spearman correlation matrix between all analyzed, soluble T cell activation/exhaustion markers, age, and IFN-γ/IL-2 responses to spike peptides**. (A) Spearman correlation coefficients (r) and (B) Spearman correlation p-values. Significant p-values (p<0.050) are marked in green. Combined 18 months data for younger and older adults infected and not infected between 12 and 18 months (I+N) are included in the analysis.

The plasma levels of soluble T cell activation/exhaustion markers were measured in 65 older adults (median 85 yo) and 34 younger adults (median 39 yo). However, sCEACAM was measured only in 18 older and 19 younger adults for which we also had cellular data. The expression of sTIM-3, sCD25, sCEACAM1 and sLAG-3 were relatively stable over the time course, from pre vaccination to 9 months (Fig. 5A and 5B, sCEACAM1 and sLAG-3 not shown). There was higher expression of sTIM-3 and sCD25 in older individuals, regardless of sampling time/vaccination status and vaccine response (Fig. 5A and 5B, respectively). The expression of sTIM-3 correlated strongly with age (Fig. 5C and supplementary Fig. 3). A similar, albeit more moderate pattern was seen for sCD25, but not for sCEACAM1 (Fig. 5C and supplementary Fig. 3). There was a significant correlation between sLAG3 levels at day 0 and the age of the vaccine recipient.

IFN-γ levels, and to a lesser degree IL-2 producing cells correlated negatively with age, particularly after boosting at 12 m. At 18 months there was no significant negative correlation between cytokine producing cells and age. However, when combining the responses at 18 months from persons with and without breakthrough infections, there was a negative correlation between IFN-γ and IL-2 positive cells at 18 months versus age (supplementary Fig. 3).

There was a moderate negative correlation between sTIM3 and IFN-γ FluoroSpot responses at all time points after day 0, except at 18 months (Fig. 5C). This negative correlation was strongest for IFN-γ after boosting (12 m). A similar but slightly weaker and less consistent negative correlation was also found between sCD25 and IFN-γ. At 18 months, IFN-γ levels correlated negatively with sTIM-3 and sCD25 when combining data from persons with and without breakthrough infection (supplementary Fig. 3).

The sTIM3 and sCD25 levels observed at all time points versus IL-2 responses at 3 weeks and 12 months (after first dose and after boosting) correlated weakly but significantly.

**Supplementary Fig. 4:**
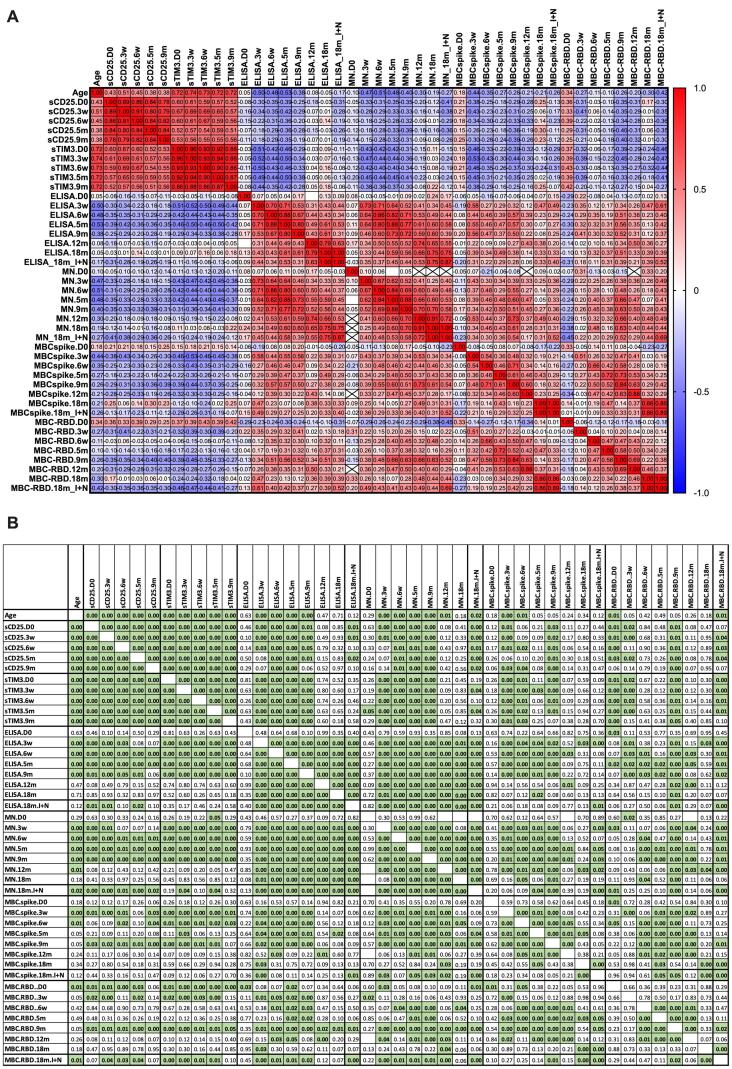
**Spearman correlation matrix between soluble T cell activation/exhaustion markers sTIM-3 and sCD25, age, and humoral responses**. (A) Spearman correlation coefficients (r) and (B) Spearman correlation p-values. Significant p-values (p<0.050) are marked in green. Combined 18 months data for younger and older adults infected and not infected between 12 and 18 months (I+N) are included in the analysis.

Furthermore, there was a weak-moderate negative correlation between antibody responses (ELISA and MN titers) at three weeks and up to and including 9 months versus age, sCD25 and sTIM-3 (supplementary Fig. 4). However, at 12 and 18 months, the only significant negative correlation was between 12 months MN titers and age. We found a consistent correlation between 3 weeks to 9 months MBC responses to spike and subsequent early, 3 weeks to 9 months antibody responses, while there was a strong and consistent correlation between 9 months MBC responses to spike and RBD versus subsequent MN titers after boosting (12–18 m).

**Supplementary Fig. 5:**
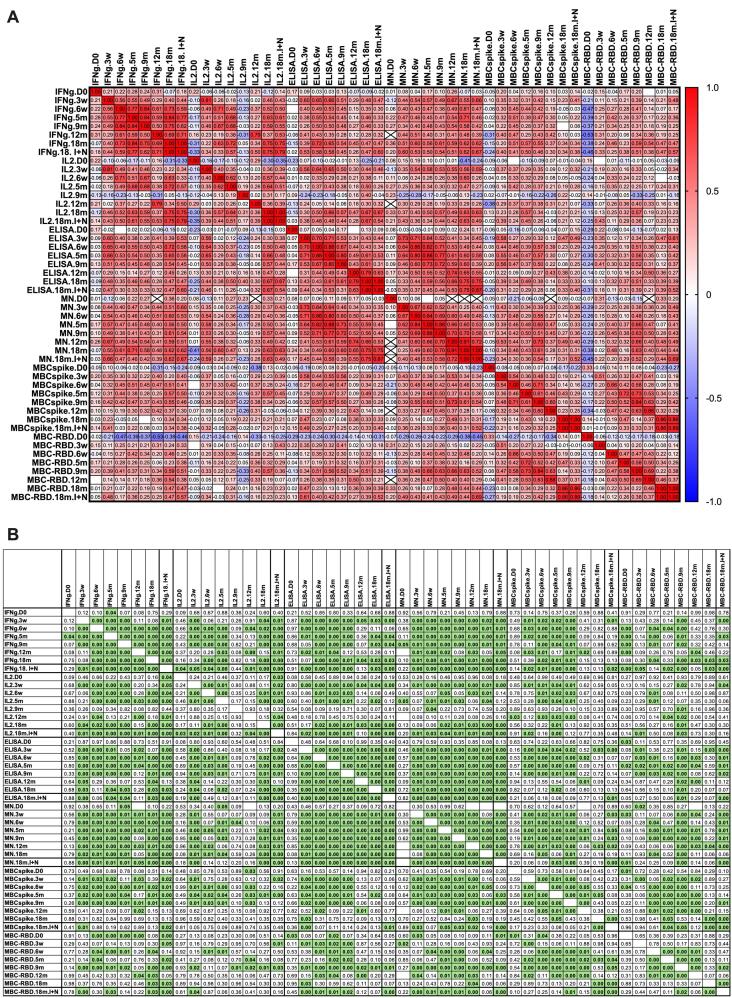
**Spearman correlation matrix between IFN-γ/IL-2 responses to spike peptides and humoral responses**. (A) Spearman correlation coefficients (r) and (B) Spearman correlation p-values. Significant p-values (p<0.050) are marked in green. Combined 18 months data for younger and older adults infected and not infected between 12 and 18 months (I+N) are included in the analysis.

Overall, there were significant positive correlations between humoral and cellular responses to spike from 3 weeks up to and including 18 months with strongest and most consistent correlations between IFN-γ ELISpot and MN titers (supplementary Fig. 5).

## Discussion

In this study, we performed a comparative analysis of the long-term humoral and cellular immune responses elicited by three doses of the mRNA COVID-19 vaccine in a group of younger adults (median age 39) and older individuals (median age 86), all of whom had not been previously infected with SARS-CoV-2 prior to vaccination. Significant differences in the immune response were observed between the two cohorts following the administration of the COVID-19 vaccine. In general, older individuals elicited weaker neutralizing antibody, spike specific antibody, MBC and T cell responses in comparison to younger vaccinees. The discrepancies between the two age groups were, however, more apparent following the first two doses of the vaccine. Following the booster dose, the differences in the antibody responses and to some degree the anti-spike MBC responses between the two age groups were significantly reduced. Reassuringly, antibody responses remained robust for up to 18 months, and both groups were able to elicit antibody responses to breakthrough infections between 9 to 18 months post vaccination. A significant difference between the two age groups was the lack of an IFN-γ and IL-2 producing T cell response in the older individuals after the booster dose. The lack of a robust, long-term T cell response in older adults following the booster dose suggests that the regimen of boosting with the same vaccine may not be optimal for the oldest age group. Supporting this, a recent study by Dallan *et al*, demonstrated that a heterologous vaccination regimen consisting of a primary adenoviral vaccine (ChAdOx1-S) followed by boosting with an mRNA vaccine (BNT162b2 or mRNA-1273) induced a more sustained spike-specific CD4+ and CD8+ T cell responses in older adults compared with a prime/boost regimen using only mRNA vaccines [11].

Memory B cells provide rapid induction of neutralizing antibodies upon infection with new SARS-CoV-2 variants [23], [24]. Given that the RBD-specific MBC levels at time of boosting correlated with levels of neutralizing antibodies post-boosting, our data suggest that the boosted levels of neutralizing antibodies mainly originate from RBD-specific MBCs. We observed increased numbers of RBD-specific (and to a lesser extent spike-specific) MBC between 6 weeks and 9 months particularly in younger individuals, suggesting better clonal selection and affinity maturation of RBD-specific MBC in this cohort in accordance with findings after SARS-CoV-2 infection and vaccination [25], [26]. Conversely, the poor MBC and antibody response in older adults may in part reflect limited T follicular helper cells and germinal center responses and reduced receptor repertoire and B and T cell plasticity that have been observed in older individuals [27], [28]. Following the booster dose, the elderly individuals elicited durable antibody responses that are comparable in magnitude with subjects that have acquired immunity through both natural infection and vaccination (hybrid immunity). Earlier studies show that boosting with a mRNA vaccine encoding the Wuhan spike-protein can effectively activate antibody responses against both the ancestral and variant strains, thus offering protection against severe disease and hospitalization [29], [30]. While mRNA booster vaccination expands the breadth of the antibody responses [25], [29], studies have indicated that the recall MBC response is dominated by cross-reactive antibodies targeting the ancestral strain [31], [32]. Boosting with variant-specific vaccines is therefore recommended to enhance the responses against emerging variants of concern [33], [34]. Interestingly, our previous findings in the same study cohort revealed that the administration of a booster dose enhanced the antibody responses directed towards the conserved spike S2 domain, particularly among older individuals [35]. Additionally, this antibody response demonstrated a correlation with the neutralizing response against SARS-CoV-2, highlighting the potential of the S2 domain as a target for vaccines that are more effective across the age groups.

A significant finding in this study was the absence of an IFN-γ and IL-2 producing T cell response in older adults following the booster vaccine dose. The loss of cytokine production is a hallmark of exhausted T cells, with IL-2 production and cytokine polyfunctionality being lost early, followed by TNF-α and IFN-γ [36]. Earlier studies have also noted weaker T cell cytokine (IFN-γ, IL-2, TNF-α) responses after two doses of the mRNA vaccine in older adults compared to younger individuals [10], [37]. It remains unclear whether repeated exposure to antigens leads to T cell exhaustion in the context of COVID-19 vaccination [38], [39], [40]. In the present study, we show increased levels of T cell exhaustion/activation marker sTIM-3 and activation/Treg marker sCD25 in relation to age. Moreover, we found an inverse correlation between sTIM-3/sCD25 and both INF-γ-expressing and IL-2-expressing T cells. This suggests that persistent T cell activation/exhaustion or regulatory T cells may have contributed to the impaired spike specific T cell response to vaccination in the elderly cohort [41], [42], [43]. While CD25 and TIM-3 are predominantly expressed by T cells, with levels correlating with cell membrane-bound counterparts [44], [45], the secretion of particularly sTIM-3 by other innate immune cell types cannot be excluded, as has been reported previously [46]. Additionally, elevated sCD25 and sTIM-3 responses are associated with various viral infections [47], [48], [49], while recent studies show that sCD25 promotes the expansion of antigen-experienced regulatory and memory T cells [50], [51]. Collectively, these observations suggest that higher plasma levels of sCD25 and sTIM-3, particularly in the adult population, may lead to creating an immunosenescent/exhausted environment that could impact the subsequent cellular responses to vaccination.

Our data further demonstrated that T cell responses to breakthrough infection, following the booster dose, were significantly weaker in the older cohort compared with the younger vaccinees. Interestingly, we found no indication that the individuals with breakthrough infections had lower antibody titers prior to infection (12 months data). However, we received PBMCs from only two of the older adults at 12 months who later experienced breakthrough infections, making it difficult to interpret the influence of cellular responses on breakthrough infections in the elderly. Overall, the failure to induce an antigen-specific T cell response in the elderly is particularly concerning considering the protective functions of T cell during SARS CoV-2 infection [52], [53]. Additionally, diminished T cell responses in older individuals can result in failure to generate an adaptive immune response, thus increasing the susceptibility to severe COVID-19 disease [53]. Comparison of SARS-CoV2-specific T cell responses across different studies is challenging due to different methods to assess these responses. In our study, we employed the highly sensitive FluoroSpot method to measure IFN-γ and IL-2 released by T cells. While FluroSpot is a highly sensitive method, alternative methods such as flow cytometry may offer an advantage of detecting low frequency cells. Previous studies using flow cytometry have demonstrated that second and third mRNA vaccine doses effectively enhance both CD4 and CD8 T cell responses targeting the SARS-CoV2 spike protein in both older adults and younger individuals [14], [54]. Using diverse immunological assays with different levels of sensitivity and capability to assess functionality and phenotype, can result in varying interpretations of the SARS-CoV-2T cell immunity [55], [56]. Thus, it is crucial that standardized assays are employed when comparing immune responses across various vaccine clinical studies [55], [56], [57].

A limitation of this study is the small sample size of the cohort that was infected with SARS-CoV-2 prior to vaccination. Additionally, older adults had more co-morbidities and had higher usage of immunosuppressants, both of which may impact the vaccine- and infection- induced immune responses, as has been reported in previous studies [58], [59].

Overall, the longitudinal design of this study allowed for a comprehensive analysis of both the magnitude and quality of the vaccine-induced immune responses over 18 months. The findings demonstrated the importance of booster covid-19 vaccinations, particularly among the elderly population that may have diminished immunity due to age-related factors or other underlying health conditions. While the third dose boosted the antibody responses, it did not increase the cellular response in the elderly cohort. The use of heterologous vaccination, different dosing or the use of adjuvants are potential alternative strategies to improve the immune responses elicited by the COVID-19 vaccines, especially in older populations that are at greater risk of severe COVID-19 outcomes [11], [39], [60], [61].

## Bergen COVID-19 Research group

Lena Hansen, Jan Stefan Olofsson, Helene Heitmann Sandnes, Kristin Risa, Olav Ervik, Siri Øyen, Lisbeth Mørk, Sarah L. Lartey, Håkon Amdam, Therese Bredholt Onyango, Mai-Chi Trieu, Nina Urke Ertesvåg, Elisabeth Berg Fjelltveit.

## Funding

The Influenza Centre is supported by the European Union (EU IMI 115672 FLUCOP, IMI 2 101007799 Inno4Vac, H2020 874866 INCENTIVE, H2020 101037867 Vaccelerate), Helse Vest (F-11628, F-12167, F-12621), the Trond Mohn Stiftelse (TMS) (TMS2020TMT05), the Ministry of Health and Care Services, Norway; the Norwegian Research Council Globvac (284930); the Faculty of Medicine, University of Bergen, Norway; and Nanomedicines Flunanoair (ERA-NETet EuroNanoMed2 i JTC2016). Research unit for health surveys/Forskningsenhet for helseundersøkelse received support from TMS.

## CRediT authorship contribution statement

**Geir Bredholt:** Writing – original draft, Visualization, Investigation, Formal analysis. **Marianne Sævik:** Writing – review & editing, Resources, Formal analysis. **Hanne Søyland:** Writing – review & editing, Resources, Formal analysis. **Thor Ueland:** Writing – review & editing, Resources, Investigation. **Fan Zhou:** Writing – review & editing, Investigation, Formal analysis. **Rishi Pathirana:** Writing – original draft. **Anders Madsen:** Writing – review & editing, Investigation. **Juha Vahokoski:** Writing – review & editing, Resources. **Sarah Lartey:** Writing – review & editing, Investigation. **Bente E. Halvorsen:** Writing – review & editing, Formal analysis. **Tuva B. Dahl:** Writing – review & editing, Formal analysis. **Mai-Chi Trieu:** Writing – review & editing, Investigation, Formal analysis. **Kristin G.-I. Mohn:** Writing – review & editing, Resources, Project administration. **Karl Albert Brokstad:** Writing – review & editing, Project administration. **Pål Aukrust:** Writing – review & editing, Resources, Investigation. **Camilla Tøndel:** Writing – review & editing, Project administration. **Nina Langeland:** Writing – review & editing, Supervision, Project administration, Conceptualization. **Bjørn Blomberg:** Writing – review & editing, Project administration, Conceptualization. **Rebecca Jane Cox:** Writing – original draft, Supervision, Project administration, Funding acquisition, Conceptualization.

## Declaration of competing interest

The authors declare that they have no known competing financial interests or personal relationships that could have appeared to influence the work reported in this paper.

## Data Availability

Data will be made available on request.
